# Parenting a Child with Phenylketonuria (PKU): an Interpretative Phenomenological Analysis (IPA) of the Experience of Parents

**DOI:** 10.1007/s10897-018-0227-7

**Published:** 2018-02-21

**Authors:** Katie Carpenter, Anja Wittkowski, Dougal J. Hare, Emma Medford, Stewart Rust, Simon A. Jones, Debbie M. Smith

**Affiliations:** 1 Division of Psychology and Mental Health, University of Manchester, Manchester, UK; 2 Greater Manchester Mental Health NHS Foundation Trust and Manchester Academic Health Science Centre, Manchester, UK; 3 Division of Psychology and Mental Health, School of Health Sciences, Faculty of Biology, Medicine and Health, The University of Manchester, Brunswick Street, Manchester, M13 9PL UK; 4 School of Psychology, Cardiff University, Cardiff, UK; 5 Manchester Centre for Genomic Medicine, Central Manchester University Hospital NHS Foundation Trust, Manchester, UK

**Keywords:** Phenylketonuria, Parents, Experience, Interpretative phenomenological analysis, Qualitative

## Abstract

Phenylketonuria (PKU) is a rare inherited metabolic disorder which can cause neurological damage if left untreated. PKU is identified through newborn screening in developed countries, and treatment begins immediately to prevent these severe consequences. When a child is diagnosed, parents must assume immediate responsibility for the management of PKU and prevention of neurological damage. Quantitative studies have identified significant psychosocial stressors for parents, but little is known about how the parents experience this process. This study aimed to explore the experiences of parents of children with PKU under the age of two. It is the first study to examine these experiences in this way. Seven parents were interviewed about their experiences, and interpretative phenomenological analysis was used to analyse the data. Three main themes were identified: *control*, *striving for normality* and *acceptance of PKU as a continuum.* Links between the themes and processes underpinning the results were explored with relation to existing literature and theories from a clinical psychology perspective. The role of acceptance of PKU was central to the parent’s experiences. Clinical implications and suggestions for further research are discussed.

**Electronic supplementary material:**

The online version of this article (10.1007/s10897-018-0227-7) contains supplementary material, which is available to authorized users.

## Introduction

Phenylketonuria (PKU) is an inherited metabolic disorder (IMD) with a prevalence of 1 in 10,000 births (National Society for Phenylketonuria 2014; Williams et al. 2008). People with PKU cannot metabolise phenylalanine (phe), an amino acid found in most protein sources. Phe accumulates in the brain and blood, resulting in permanent neurological damage and concomitant intellectual disability and epilepsy. This can be averted through a strict life-long regimen of a phe restricted diet and amino acid supplements (National Society for Phenylketonuria 2014; Al Hafid and Christodoulou 2015). Management of PKU places significant demands on parents with supervision of the child’s nutritional intake, ongoing medical appointments and regular blood tests. Parents of children with IMDs report significant burden (Gramer et al. 2014) and challenges include time constraints, stress and restrictions on social life (Bilginsoy et al. 2005; Eijgelshoven et al. 2013) along with emotional, mental and interpersonal stress (Cederbaum et al. 2001; Packman et al. 2007). In particular, there are stressors relating to dietary provision, the threat of metabolic crisis, coping with uncertainty, managing life transitions and struggling for improvement in care (Khangura et al. 2016; Medford et al. 2017a; Read 2003; Storch et al. 2008; Zeltner et al. 2014).

When their child is diagnosed with an IMD, parents are expected to adapt and cope (Abidin 1990), integrating generic parenting with specific tasks related to their child’s disorder, whilst managing their own emotions and adjusting to their child’s diagnosis and prognosis (Turner-Henson et al. 1992). Actual and perceived high burdens of care are associated with parental stress (Calderón et al. 2011; Cousino and Hazen 2013), and parents of children with chronic conditions experience a high burden of care together with elevated levels of emotional distress and poorer adjustment (Cadman et al. 1991).

Research in parenting stress in PKU has yielded mixed findings. Some parents report significant care demands, whilst some report similar or even lower levels of stress and higher quality of life when compared to parents of healthy children and parents of other inherited metabolic disorders (Kazak et al. 1988; Ten Hoedt et al. 2011). This is complicated by an age-related finding that parents of younger children with PKU reported impaired quality of life compared to parents of older children (Fidika et al. 2013; Ten Hoedt et al. 2011). Parents of children with PKU report significant emotional challenges early on in their child’s life, including grief and trauma reactions alongside caring for their new baby (Awiszus and Unger 1990; Lord et al. 2008).

There has been recent quantitative research into the impact of caring for a child with PKU and impact on parents’ quality of life (Bosch et al. 2015). They found that the highest impact scores were ones measuring the emotional impact of PKU and its management and that there was a greater impact on parents of younger children. Currently, although research suggests that early childhood could be a particular challenge for parents of children with PKU, there is a paucity of research exploring their lived experiences, the processes parents go through in adjusting to and coping with these challenges and how they make sense of their experience. The present study aimed to explore the lived experience of parenting a child with PKU in the first 2 years.

## Method

### Participants and Recruitment

Ethical approval was granted by University and NHS Ethics Committees (ref 15/NW/0454). Purposive sampling was used with parents and caregivers of children with PKU. Study design, protocols and participant materials were discussed with and approved by the National Society for Phenylketonuria (NSPKU 2014). Parents with children under 2 years old were recruited from three PKU clinics in the North of England as part of a wider study (Medford et al. 2017a, b). After assessment by the clinical team, parents were excluded if there were other significant family stressors (e.g. other significant care giving responsibilities) or did not speak English. Eligible participants were sent postal information packs. In addition, advertisements were placed in waiting rooms, Facebook groups and NSPKU newsletters. Parents who wished to participate returned consent forms, and interviews were booked accordingly.

### Interview Protocol and Procedures

Parents were interviewed in their own homes using a semi-structured interview schedule of four broad areas (see Appendix 1), which allowed for probing and further exploration of arising areas of importance for participants (Smith et al. 2009). Interviews were conducted, audio-recorded and transcribed verbatim by the first author (KC). Parents of the same child were interviewed separately. The interview schedule was developed through reviewing extant literature and discussion with clinicians and was approved by the NSPKU 2014). Four areas were identified as prompts for parents: experience of the diagnostic process, processes of parenting, challenges and coping and support.

### Data Analysis

Data were analysed using interpretative phenomenological analysis (IPA; Larkin et al. 2006; Smith 1999) according to Smith and Osborne (2008) guidelines so as to explore both how people make sense of their experiences and the significance they attach to these experiences (Smith et al. 2006). Analytic diaries were kept by the first author to enable reflection on pre-conceptions and ideas. Two researchers (KC and DS) undertook each stage of data analysis independently. All transcripts were re-read several times, and each researcher’s reflections on the transcripts noted and bracketed to ensure that data-led interpretations were derived. Each transcript was analysed line by line (descriptive coding) to elicit key meanings, understandings and matters of importance to the participants. Linguistic and conceptual comments were made on the data. Frequent patterns in responses were identified firstly within individual transcripts and then across all transcripts to develop themes (subordinate themes). Relationships between themes were drawn out by two researchers (KC and DS) and, following discussion, organised to provide a detailed narrative of the analysis. Each stage was discussed by the two researchers to ensure that interpretations were plausible, coherent and grounded in the data. Any disagreements were resolved through discussion of the text and reasoning given by each researcher, taking into account their subjective position. The final interpretation was corroborated by anonymous data excerpts and finalised by the research team.

### Reflexive Positioning

As qualitative researchers bring their own preconceptions, expectations, knowledge and experiences to the research process, reflexivity is an important part of qualitative research and replicability (Smith et al. 2009). Although not a parent herself, the first author (KC) had 2 years’ experience of working with parents of children with intellectual and developmental disabilities, including post-diagnostic support for parents whose children had been diagnosed with autism. The research team included a health psychologist (DS) and a clinical psychologist (AW), both of whom were parents as well as researchers into parenting. The interpretative process is likely to have been influenced by these experiences and knowledge and experiences of using and applying psychological models and understanding experiences from a clinical psychology perspective. All other co-authors (SJ, SR, DJH) have clinical experience in this field and approved the final analysis.

## Results

Eighteen research packs were sent out. One participant (6%) declined to participate. Two parents (11%) consented, but they were unable to be contacted using the details provided. No information was available for participants who did not respond (44%). Seven (39%) of the invited participants were interviewed; six mothers and one father consented to participate and were interviewed in their home. This included the mother and father of one child. In total, caregivers of six children with PKU were represented. Median length of interview was 111 min (range 62–137 min). All participants were white British, married and living with their partner and child(ren). The sample size of seven was appropriate for IPA studies (Smith et al. 2009), with theoretical sufficiency being obtained within this sample, which is comparable to other IPA studies of parents of children with chronic conditions (Schweitzer et al. 2012; Smith et al. 2006; Smith et al. 1999).

Three superordinate themes, *control*, *striving for normality* and *acceptance of PKU as a continuum* and 11 subordinate themes were identified. The three superordinate themes were closely associated with each other; first parents described a process of gaining control over the management of PKU to ensure their child was healthy and to prevent neurological damage, then establishing a “normal” life for their child to minimise the impact of PKU on the child. The following stage of acceptance of PKU was described as a continuum, with parents’ experiences falling into acceptance or non-acceptance of different aspects of PKU. The potential impact of parents’ experiences and attitudes on their child’s adjustment in the future is considered in the discussion.

**Fig. 1:**
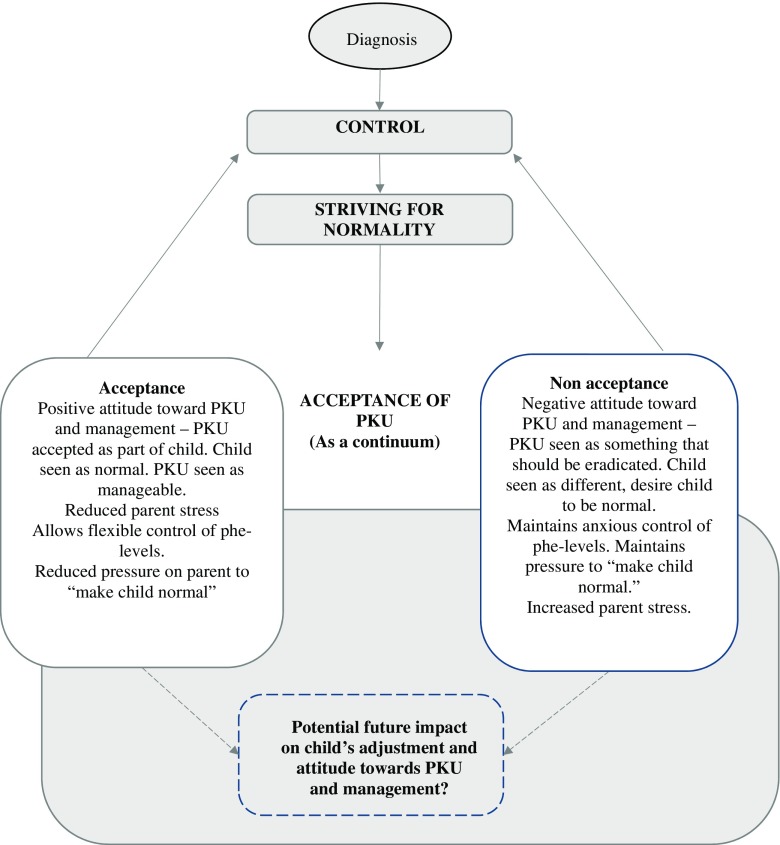
Diagrammatic representation of superordinate themes

Figure Fig. 1 illustrates the themes and processes. Illustrative quotes can be found below each theme described in detail in the next sections. Pseudonyms have been used to protect the participants’ identity.

### Superordinate Theme 1: Control

Parents reported that healthcare professionals appeared to expect them to gain immediate control of their child’s blood phe levels, irrespective of their emotional reaction. By maintaining this strict dietary control, the requirement of control manifested itself in many aspects of the parents’ lives. Three subordinate themes were identified: *fears of the consequences of non-compliance*, *increased personal responsibility* and *emotional* and *social consequences of control.*

#### Fears of the Consequences of Non-compliance

All parents expressed worry about being unable to control blood phe levels due to the serious consequence of non-compliance with the dietary treatment. The threat of brain damage was ever present, especially soon after diagnosis.

*Alice: Some days would be good because you’d think “It’s fine, it’s just a diet and blah de blah”… and then you have other days when you think, “Well what happens if she doesn’t stick to her diet… and what happens if we don’t…” and you know them things that go through your head… …… you don’t want your child to have any kind of brain damage, do you?*

Parents felt uncertainty about how effectively they managed the diet due to a lack of immediately observable effects of raised phe levels and reliance on feedback from blood tests as a marker of success. This fear was exacerbated when confronted with dietary challenges, such as their child not wanting to eat or refusing supplements.

*Nina: I just can’t help thinking, when his bloods are high… there’s got to be something happening to his brain, it’s got to have some sort of impact, that toxic build up…… I just don’t know… so yeah, it’s scary.*

#### Increased Parental Responsibility

Parents described feeling overwhelmed by the demands of care that were placed on them immediately following diagnosis. All parents reported feeling some guilt for passing down a genetic disorder.

*Jemima: I felt so many emotions… I felt… my main one was guilt… like… like I felt guilty… once I’d found out that it was genetic.*

Parents assumed exclusive responsibility for preventing neurological damage. Fear of relinquishing control by allowing others to care for their child reduced the amount of support they were able to access. Support from family members was a source of both relief and further stress depending on the perceived competence of the care giver to care adequately for the child. There was a reluctance to allow or burden others to care or prepare food for their child unless parents were sure they were capable of managing the diet, which all parents reported caused them to delay putting their child into nursery care or daycare.

All parents reported that they adjusted their working patterns, the majority sent prepared food along with the child and all had invested time teaching relatives and professionals before they allowed them to care for their child and child’s diet. Parents described this as necessary to reduce the burden on others and to alleviate their own anxieties about handing over control of their child to someone else.

*Amy: I think I’m quite… I like to take ownership of it… so I suppose the food diary helps me with that… erm… I like to be in control of it… so it’s very much reliant on me… like… [husband] doesn’t really take… much involvement at all… in… the management of his he… his diet… but… I suppose that’s just how I’ve dealt with it… I've taken it on control… and… I’m sort of in charge…*

#### Emotional and Social Consequences of Control

Despite good self-reported treatment adherence, parents experienced continued pressure, mostly self-imposed and partly imposed by healthcare professionals, to maintain good blood phe levels. Social situations involving food increased parental stress, anxiety and the need for “constant vigilance” to ensure their child did not eat restricted food. Parents felt varying levels of need to “protect” their child from situations where restricted food was accessible. All parents expressed guilt for either feeding or denying their child certain foods.

*Amy: I remember the first one, making it and just thinking, how can I give my child this? How can I… feed my child something that smells so bad, something that I wouldn’t even dream of drinking?*

All parents reported some degree of frustration and anxiety when their child did not eat “planned” food, for example if they had planned to give a certain number of protein exchanges for that meal and the child refused to eat it and stress about recalculating exchanges if their child had eaten some of the meal but required alternative food. Some parents described feelings of failure if they were unable to provide varied, low protein food that their child would eat, and all parents felt some degree of this when blood results were high.

*Emma: Well, I’m on the phone crying, I get her on facetime (gesturing to mum)… [and say] “You tell him mum, will you speak to him mum…” and I don’t want him to see that I’m upset, and frustrated and that I can’t do it.. and then I feel like I’m… as a parent I’ve let him down.*

Strains on relationships were increased when this happened, because parents doubted themselves and suspected others (partner, family members, and professionals) of giving their child something they were not supposed to have by accident when levels were high. This resulted in reduced availability and utilisation of support, which increased the burden for parents. Parents who could confidently leave their child with others felt less anxious and found time to relax.

*Ben: I find myself at work thinking… hmm I wonder what’s going on now, I wonder if she’s had her food, I measured it out but I wonder if she's given them or.. you know… has she given her some milk, or something like that… some cow’s milk to settle her. You don’t know. You just think because they aren’t there with us when we’re talking to the doctors and stuff like that… it does… it’s a challenge for me to try and wipe that out.*

The necessities of being organised and planning in advance to meet the demands of managing PKU meant that parents felt that they lost the joy of spontaneity and were less able to engage in social activities (such as eating meals out). All parents were concerned that the requirements of following the diet would cause others to judge them due to a lack of understanding about PKU and its management.

*Alice: We’ve got to watch haven’t we, what she’s doing, whereas if people have no understanding of it at all, they’ll just… they’ll think I’m some mad woman, you know like… going… if she starts picking something up, I’m like “NO YOU CAN’T EAT THAT” thinking I’m some kind of crazy woman, who doesn’t let her kid eat… certain things.*

### Superordinate Theme 2: Striving for Normality

Parents did not want PKU to define their child or them as a family. The next step, following maintaining control over blood-phe levels and the management of PKU, was to minimise the impact of PKU on the child to ensure that they were able to live a normal life. The reported impact of PKU on the life of parents was varied. All parents wanted to ensure that PKU did not dominate their child’s life, although this could have a paradoxical effect by dominating the parents’ life. Four subordinate themes were identified: *a different healthy child*, *fear of child feeling different*, *effortful creation of a normal life* and *achievement*.

#### A Different, Healthy Child

Most, but not all parents felt their child was normal with a different diet and that PKU was only a small part of their child. Parents reflected on their child occupying a “liminal space” between being healthy and ill (Diesen et al. 2015) due to their child having a serious disorder but not being ill or having anything wrong due to the asymptomatic nature of well-managed PKU.

*Amy: Obviously we understand the seriousness of it, but it’s so important for me and [husband] that we don’t let it take over… don’t let it define her…. it’s just something that she has, it’s not… doesn’t make her who she is.*

#### Fear of Child Feeling Different

Parents described that PKU was invisible until occasions where food was present. As food was viewed as an important part of social life, parents expressed concerns about their child fitting in, being treated differently compared to others, or that their child would feel different from others or be excluded.

*Jemima: I worry about [child] going to high school… I worry about him being in primary school and being picked on… food is such a social side of your life these days… everyone goes out for tea… and stuff like that, and it breaks my heart to think, you know… that’s gonna be difficult for [child].*

Concerns were present and future based; parents considered both the social impact of PKU later in their child’s life (e.g. at parties, school) and how they would cope with the increased demands of minimising this impact. Parents feared their child missing out on opportunities because of their PKU and felt determined to prevent this from happening. Parents were concerned their child would feel different within the family. Parents were navigating challenges, such as eating together, considering the impact on siblings, and striking a balance that was fair to their child and did not affect family members.

*Nina: But it was hard to accept that he was going to have a different… kind of life, if you like… and then I kept thinking really far in the future like… thought about when he wants to go to parties… and… other kids are eating cake and… he can’t join in… and you know… when he's a teenager and they all go to McDonald’s, and he… he’s just got to sit there with a little bag of fries…*

#### Effortful Creation of a Normal Life

All parents felt that PKU should not restrict their child from any normal activities. Fear of their child not being or feeling normal motivated parents to strive to prevent or minimise any feelings of “difference” arising from the diagnosis and management of PKU. Parents felt responsible for minimising the impact of PKU for the child. It was important to parents that the food their child ate looked similar to food others were eating.

*Amy: That’s the key thing that it’s helpful with, and sharing recipes, that’s helpful. And lots of people do PKU and non-PKU versions and show you things you can eat together.*

Family routines were changed to ensure the child did not feel left out (e.g. not eating food the child could not have in front of them, not eating out in restaurants). Parents experienced moment of sadness in situations that highlighted that their child was different, despite their best efforts, which motivated them to work harder to reduce this difference and their sadness.

*Amy: But… and a lot of the time as well, [husband]’s mum and dad have her quite a bit, so at a weekend we’ll either wait until she's in bed to have our tea, because we don’t want to eat in front of her because we feel bad, or she’ll go round there and we’ll have our tea early, and we’ll go and pick her up, so she’ll be there for an hour or so whilst we eat our tea.. just so we’re not eating in front of her.*

However, difficulties in acquiring low-protein foods were considered a barrier to achieving “normality”. Making or buying food that looked normal placed a burden on time, resources and finances. In order to make their child feel normal, included and keep them healthy, parents were required to give their child “special treatment”. These attempts at minimising the impact of PKU on the child increased the impact on parents.

*Ben: … give [child] food that reflects or looks like something that her friends are having… so when she does get to school, it’s not obviously different to what everyone else is having… therefore it goes under the radar.*

#### Achievement

Alongside the challenges of providing a normal life for their child, parents described many positive aspects, despite the potentially severe consequences of PKU when insufficiently managed. Seeing their children develop normally and do things that other children did and seeing other children with PKU doing normal things gave parents hope and reassurance that PKU had a minimal impact on their child’s life, both at present and in the future.

*Lucy: …and I can see him doing exactly the same as the other children… and they don’t even know he has PKU… but he’s still… just normal… you know… I find that really positive… erm… finding… you know, going to a restaurant and finding something on a menu… that they do have something that he can have, I find that really… I find that like a happy moment… erm. Finding something in the supermarket that he can have, that’s like a really positive moment… like… I've just recently found, [name of company] do some white chocolate buttons that he can have… and it’s like, woo! So, I buy loads… erm… just little things like that I suppose, make me… give me a bit of a boost.*

Parents reflected on their ability to overcome challenges, such as taking the blood, cooking, going on holiday and returning to work and to integrate these aspects of PKU management into their family life.

*Jemima: To watch him eat something I’ve cooked is great….. cause it’s such a major thing I think… erm… yeah.. Because it’s all I’m ever thinking about… trying to think of new things for him and… Things he might enjoy… then I make something and he really likes it, and I think “Yeah, I’ve cracked it. I’ve done it. I’m doing a good job.*

Despite challenges, parents felt that the effort required to create a normal life was worthwhile and they valued reassuring evidence that they were doing a good job. Parents described that seeing their child develop like any other child and enjoying the food that they had cooked made their considerable efforts meaningful. Most parents felt proud of their increasing confidence and competence in managing the demands of the diet and treatment and reported that the stress they experienced was not pervasive.

### Superordinate Theme: Acceptance of PKU as a Continuum

Acceptance was the next stage once parents had successfully gained control of phe levels, minimised the impact on the child, and seen for themselves that PKU did not have an overwhelming impact on their child or their family. Parental acceptance of PKU was described as a continuum that parents could move along as they faced new challenges. Parents described themselves as at different points along this continuum as represented by the following five subordinate themes: *acceptance of diagnosis and management*, *lack of knowledge*, *understanding and information, support from others*, *becoming the expert* and *gratitude.*

#### Acceptance of Diagnosis and Management

Parents gave almost identical accounts of their feelings at the diagnosis, including hope that the results were wrong, shock, anxiety and sadness that their apparently healthy child had something wrong with him or her.

*Nina: I’m not depressed… I’m just sad, because of my baby, but not in a depression state… I’ll get over it… but I've got to come to terms with it, because it’s different and… you know… it was a big shock…*

Parents expressed the need to come to terms with the diagnosis, although there were varying degrees of acceptance among parents. Some saw PKU as a hated or undesirable thing existing within their child. They wished that their child did not have the disorder or that it could be eradicated and were constantly hoping for a cure or radical improvements in treatment in order to make their child normal.

*Emma: Well, I ask about it every time I go. About this [medication]… and [the] doctor explained to me that [child] can have the [medication] if he wants on a trial for 6, 7 years… and his exchanges can go up from 13 to 30 a day. It’s massive. So he says, when he explained it to me… if he wants it he can possibly go on it… but do you want him to grow to the age of 9 eating completely normal, and then at 9 having to say you can’t have it no more, it was just a trial, and now you’ve got to have your prescription food and go back to the way you were.. because they can’t offer it on prescription.*

Other parents accepted PKU and its management as only a small part of their child and their life and embraced what they had to do to manage PKU rather than try to remove it.

*Alice: Obviously if she… I wish, no I don't wish she didn't have it, but it would be easier if she didn't have it… wouldn't it like.. For the worry and stuff, but it's not I don't… think, I don't think… oh I hate PKU and grrr… Like I said she's my little girl and I'll do anything for her…. and so I am doing… you just get on with it…*

Once parents accepted the management regimen and that blood levels were not always completely within their control (e.g. when the child was ill or teething, or would not eat), they were able to adopt a flexible approach to the dietary management of PKU which reduced the stress they felt when their child was not eating. Conversely, in this study, high levels of stress were maintained in parents who were less accepting.

#### Lack of Knowledge, Understanding and Information

All parents reported a lack of knowledge and understanding about PKU from midwives, other healthcare professionals (GPs, chemists) and the general public. Lack of knowledge from midwives was deemed to have contributed to more intense reactions of fear and shock at the diagnosis, as all parents reported a false sense of security at the heel prick test, either by midwives who stated that results were unlikely to come back as abnormal or because they had other children whose tests had come back clear.

*Lucy: This one midwife came round when he’d just come out of hospital… and… she said to me, “It can be really hard when your child’s diagnosed with a life threatening condition”… and… thankfully at that point I knew it wasn’t a life threatening condition… but., that was kind of like… it’s hard when you know, when professionals don’t obviously know anything about it… and when they say things like that… that… that… at the time was just kind of like… I didn’t really know what to say…*

Friends, family members and the general public were reported to frequently misunderstand PKU; parents were often asked if it was like an allergy, or if their child would grow out of it. A lack of knowledge was viewed as having potentially threatening consequences (e.g. someone giving their child something with protein by accident).

*Ben: So without doubt the diagnosis of PKU was the worst part… and we envisage it being the worst part as well… cause for us… there wasn’t someone on the phone to say, “Look this is what she's got, this is what it, this is how it’s going to affect you… by the way, don’t worry it’s manageable…” we didn’t have that. We didn’t have that point. We were just told that “This is something that is very very rare, that’s all we can tell you.”*

Some parents felt that the “mixed messages” about PKU (treatable but serious, child is “ill” but “not ill”) was a barrier to acceptance, contributing to feelings of uncertainty and an ever present threat of symptoms and preventing them from resolving their feelings about the diagnosis.

*Jemima: We’re treated as parents with PKU… is because of the lack of knowledge of the condition we’re kind of dismissed a lot… erm… as it being not a serious condition… this is what I find frustrating as a parent.. is that people don’t take the condition seriously… it is very confusing, like… you’re being told how you should feel… a lot of the time.*

However, some parents felt that this was positive and hopeful as PKU could be treated to ensure that “illness” did not occur. Some parents reported that a perception of PKU as not serious meant that their concerns were dismissed, leading to anxiety and frustration. Some parents felt “it could be worse”, but other parents felt that PKU was a terrible disorder to have and this attitude left them feeling invalidated or that their negative feelings about PKU were unjustified.

#### Support from Others

Parents described how they mitigated the lack of information and awareness from others through contact with and support from other parents of children with PKU (mostly accessed via Facebook).

*Lucy: I joined a Facebook group, which has been amazing… just… got so much support from other people which is really good… because I think… because it’s so rare you don’t ever get chance to really see other people and see what they’re going through… so that way, through Facebook… I’m just really grateful for that support… so I joined that group and all then… suddenly you don’t feel quite so alone when you get speaking to people.*

Social networking sites were considered by all parents to be a valuable source of support for getting timely, practical advice about food and recipes, sharing experiences and getting emotional support that they felt was lacking from professionals, although every parent interviewed praised the practical advice and support they received from professionals. All parents accessed support through social networking at the point of diagnosis and were immediately contacted by parents of children with PKU who were able to offer “proof” that PKU had a minimal impact on child development and life prospects and that challenges could be overcome.

*Nina: That’s when you go to your PKU mums and say… “I’m panicking, [their phe levels] have been up for this many weeks… what’s causing it, what can I do… is it going to do him any harm…” and they all tell you the same thing… “Don’t panic.”*

*Emma: If I’m out and about and I’m in a restaurant and there’s something I don’t know, I’ll put a question on Facebook, and whoever’s out there, out of the thousand people, because they’re from America and everywhere, they’ll jump in and answer my question for me. So, I’ve got constant 24-hour-support.*

Parents valued family and friends doing their own research which enabled parents to accept help and support for their child from wider systems and created a normal life outside the immediate family environment which reinforced a wider support and acceptance of PKU (acceptance beyond parents).

*Amy: … but when the people that did lots of research, I appreciated that as well… just to show that you’ve got that support there. I did appreciate that, it made me feel… just cared for and heard.*

#### Becoming the Expert

Parents described a process of acquiring knowledge and becoming “PKU experts”. Many felt that raising awareness of PKU and educating others was the best way to help their child and could promote future advances (e.g. supermarkets and restaurants supplying low-protein foods). Some parents became “activists”, for example organising events and raising money for charity, whereas other parents raised awareness by helping people understand PKU.

*Nina: I mean I’ve kind of lived and breathed it since he was… since we found out… and you know, the dieticians said that, they said “You’ll probably become more knowledgeable about it than us, because… you know… he’s your son, and you’ll do everything you can.”*

Some parents were reluctant to discuss PKU with others; reasons included the wish not to make a big deal about PKU, finding it emotionally draining, or wanting the child to have the opportunity to tell people themselves when they were old enough. Parents experienced conflict between wanting to spread awareness and not wanting PKU to dominate their child’s narrative. Parents saw themselves in an expert role and suggested that they could be useful points of contact for other parents at the diagnosis stage.

*Emma: I’ve been into the doctors to talk about it because they didn’t know anything about it… talked to the receptionist, the two doctors… and the trainee nurse to explain to them what it is… because none of them have heard about it… and they look to me to help them… and they’re actually quite grateful that we’ve stayed with them because they’ve never had anyone with PKU, and for them it’s training.*

#### Gratitude

Parents who were more accepting of the diagnosis of PKU reflected more on the positive aspects of PKU. Many parents felt lucky that PKU is screened for, identified and treated early and that neurological damage can be prevented by diet. Their child’s achievements were described as more special, because their child’s developmental trajectory could have been radically different if PKU had not been identified and treated early.

*Jemima: Everything is so much more special when [child] does it… because of everything that was put against him you know… you can’t help but think, 60 years ago… how different my little boy would be… only 60 years ago.*

Some parents reflected on experiences of seeing other ill children which made them think how much worse things could have been had their child had a different, progressive, untreatable or life-limiting disorder.

*Nina: I hate the self-pity thing, I hate it… there’s a lot of people worse off… in that genetics department, when we go for his appointments, that kind of brings it all into perspective cause there’s some really really poorly kids in that department… and you think… gosh we’re really lucky because it’s just dietary, you know.*

Not all parents felt lucky; some felt that this attitude minimised the significant physical and emotional struggles they faced daily to manage the demands of providing the special diet for their child, whilst at the same time managing the emotional aspects of the potential threat of brain damage.

*Emma: So… you know… it’s really hard… and then you’ve got loads of people saying that you’re really lucky, but on the other hand, you’re not that lucky.*

## Discussion

This was the first study to explore the specific experience of parenting a child under the age of two with PKU, and it highlighted three processes. Firstly, parents were required to gain control over their child’s blood phe levels literally from the moment of diagnosis. Irrespective of parental feelings and emotional reactions, all parents were aware that their child’s future development depended on their ability to manage PKU appropriately. Despite anxiety about these challenges, parents soon adjusted to the requirements of maintaining the treatment for PKU, developing family routines accordingly. Following this, parents moved onto minimising the impact of PKU on their child, which required considerable effort from parents as they strived to make things as normal as possible for their child by changing the situation*.* Following successfully gaining control of PKU and witnessing their child living a normal life, most parents accepted PKU as part of their and their child’s lives and adopted a “new normal”. This is similar to the experiences of parents of children with diabetes and autism spectrum disorder, who reported positive experiences with their child’s disorder (Kayfitz et al. 2010; Sullivan-Bolyai et al. 2006).

Not all parents were accepting of the diagnosis of PKU and wished to remove PKU from their child. These parents were experiencing more intense negative emotions akin to grief reactions, in particular denial, anger and depression which come before acceptance (Kübler-Ross 2003), a finding similar to those of other parents of children with PKU and other chronic disorders (Awiszus and Unger 1990; George et al. 2007; Lowes and Lyne 2000). It should be noted that of the three mothers with more intense negative emotions, two had undergone traumatic birth events, which made their experiences difficult to attribute solely to their experiences with PKU. Trauma in the perinatal period could contribute to perceptions of the child as vulnerable, the parent as unable to cope and events as uncontrollable, which are exacerbated by the diagnosis of PKU which may be perceived as a “threat” to the development of their child (Vetrone et al. 1989). This is consistent with findings that parents with high trauma scores appeared to be constantly aware of PKU as a threat to their child or that trauma could disrupt a normal process of adjusting to or resolving the diagnosis (Lord et al. 2005).

Given the reported grief-like reactions to the diagnosis, together with the fact that parents appeared to have had little time to address these feelings (which may not have been understood as grief due to their child appearing healthy), the dual processing model of grief (Stroebe and Schut 2010) may be applicable to the parents’ experiences. Specifically, parents were immediately forced into primarily “restorative” coping (what had to be dealt with and how to deal with it), rather than the healthy oscillation between restorative and loss-oriented coping (focus on aspects of the loss itself) that ultimately contributes to resolution of grief. However, in the parents of children with chronic illness or disability, grief can be ongoing in response to continued losses or triggered by transitions in the illness (Bowes et al. 2009; Collings 2008; Lowes et al. 2004), which is consistent with the present findings of parental acceptance as a continuum. Parents of children with diabetes reported managing the treatment demands in a “robot-like fashion” (Hatton et al. 1995), suggesting restorative coping.

Parents who engaged early on in an oscillation between loss and restorative coping may have been more able to resolve their grief and reach an acceptance of PKU, as opposed to parents still experiencing impaired movement between restorative or loss oriented coping. This is proposed by the Dual Processing Model of grief (Stroebe and Schut 1999) The adaptive management of PKU may necessitate more restorative coping strategies, but importance should be placed on acknowledging and validating parental feelings at diagnosis to facilitate loss oriented coping and promote healthy adjustment and an awareness that acceptance is a continuum that parents can move along in response to their experiences. It is important to note that parents of children who received positive results at newborn screening can experience healthcare professionals as insensitive if they focus purely on medical management and neglect the parents’ emotional needs (DeLuca et al. 2011). Thus, it is important that healthcare professionals are able to acknowledge and validate parents’ emotional needs as part of the care they provide.

In addition to focusing on grief resolution, appraisals of threat or stress may influence parental acceptance of PKU in line with the stress-coping model (Lazarus and Folkman 1984) in which event-specific appraisals determine coping. Unresolved parents may thus appraise their child as vulnerable, appraise themselves as unable to cope and view the threat (PKU) as beyond their control. Although this might lead to “adaptive” coping strategies such as strict control, it may maintain anxiety or trauma reactions. Anxiety or trauma reactions can increase stress for parents and lead them to utilise disproportion strategies to minimise threat, which could result in the use of overprotective or restrictive parenting strategies that restrict the child’s emotional or social development. This is consistent with Jusiene and Kučinskas (2004) observation of maladaptive parenting strategies in parents reacting to the diagnosis of PKU with anger or guilt and with findings of Awiszus and Unger (1990) who describe coping as an attitude, not a behaviour. A lack of information and dissatisfaction with communication of abnormal screening results at the point of diagnosis increased parental distress, whilst improved information helped mitigate uncertainty and shock (Buchbinder and Timmermans 2012).

### Study Limitations

Limitations of the current study include the fact that all parents interviewed reported good treatment adherence, with no representation from parents who struggled to control blood phe levels. Only one father participated, thus limiting any inferences about maternal and paternal experiences, which are known to differ (Lord et al. 2005; Lord et al. 2008; Vetrone et al. 1989). We should also acknowledge a possible lack of international transferability due to differing healthcare services which may further impact on parents’ experiences.

### Practice Implications

Healthcare professionals find it difficult to deliver bad news and feel that they have insufficient knowledge and training regarding this (Fallowfield and Jenkins 2004; Finan et al. 2015; Warnock et al. 2010). These factors may affect the quality of information imparted when communicating positive results at newborn screening. There may also be a lack of understanding about how people cope with receiving positive screening results, which represents a role for health psychology or genetic counselling input into training professionals to deliver results. Given the influence of the information received on subsequent parental appraisal of PKU, it would be beneficial for this information initially to be communicated by someone with a comprehensive understanding of PKU who can provide parents with balanced and accurate information, has a good understanding of the emotional processes involved and is skilled in sensitive communication to reduce the emotional impact of the diagnosis. Parents have requested that professionals relaying information about newborn screening have adequate information, avoid jargon, listen carefully, encourage questions, acknowledge and validate parental distress, offer realistic reassurance and refer to specialists (Salm et al. 2012). Good quality information has been shown to provide a buffer against negative emotional reactions and facilitate better adjustment (Waisbren et al. 2003).

It is important that parental emotional well-being is considered alongside providing practical or medical advice about the management of PKU and those parents’ feelings are validated and normalised to help facilitate adjustment. Parents should be given time to discuss and process their feelings about the diagnosis with professionals such as clinical psychologists, genetic counsellors or specialist nurses, so as to acknowledge and validate parental feelings to facilitate healthy adjustment. Healthcare professionals should be aware of any experiences ante or perinatally that could disrupt emotional processing of the diagnosis (e.g. traumatic birth; Lord et al. 2008).

Acceptance of the diagnosis should be explored with parents, given the high association with parent stress, anxiety and depression (Lloyd and Hastings 2008). Dysfunctional beliefs about, for example actual and perceived controllability and level of threat could be modified using cognitive behavioural therapy to help parents develop a more balanced view of PKU and its prognosis. Parents who have not accepted the diagnosis are more likely to perceive PKU as a threat, and when in “threat neutralisation” mode may be less available to meet their child’s emotional needs, which could affect the attachment relationship (Jarvis and Creasey 1991) and parenting styles (Fehrenbach and Peterson 1989; MacDonald et al. 1997). Similarly, acceptance and commitment-based therapeutic interventions reduce depression and anxiety and facilitate better adjustment in parents of children with autism (Blackledge and Hayes 2006) and may be similarly beneficial for parents of children with PKU.

All parents interviewed wanted more contact with other parents and valued input from healthcare professionals to facilitate this contact. It would be beneficial for healthcare professionals to facilitate such meetings to allow parents to see that PKU can have a minimal impact on other children and to share experiences and expertise. Specialist healthcare professionals could provide support and training to family members and other professionals involved in the child’s care (Packman et al. 2007) to reduce parents’ anxiety about entrusting care of their child to others, which would reduce the burden on parents, increase support networks and improve parental well-being given the centrality of social support as mediator of stress, distress and quality of life in parents of children with PKU (Fidika et al. 2013, Hatzmann et al. 2009).

### Research Recommendations

Further examination of the role of acceptance in parental well-being could be explored in parents of children with PKU together with the development and evaluation of specific interventions as outlined above. It would be beneficial to explore healthcare professionals’ experience with and views on support for parents and the impact of PKU on their parenting. Given the importance of communication of the suspected diagnosis at the new born screening, examining the views of midwives who have communicated suspected PKU results to parents may also be an important focus for future research. Further studies including parents of older children with PKU would also be beneficial to identify if parenting styles and acceptance of PKU change over time.

## Conclusion

The current study examined parents’ experiences of parenting a child with PKU under 2 years of age and found that establishing control over the necessary treatment regime and minimising the impact of the disorder on the child may be independent of parental acceptance of the diagnosis. It also highlights the importance of the goal of normality and role of acceptance and how they influence parental perceptions, motivations and behaviour. The point of diagnosis has been acknowledged as a key experience in parents’ lives, which sets the context for the later process of control, striving for normality and acceptance. This has implications for improving the diagnostic process to promote better parental adjustment.

## Electronic Supplementary Material

ESM 1 (DOCX 69 kb)
