# Supplementary material for: Parenting a Child with Phenylketonuria (PKU): an Interpretative Phenomenological Analysis (IPA) of the Experience of Parents
Source: J Genet Couns. 2018 Feb 21;27(5):1074–86. doi: 10.1007/s10897-018-0227-7 (PMC6132571; doi:10.1007/s10897-018-0227-7)
Supplement: Supplementary file 1 — (DOCX 69 kb) [file 10897_2018_227_MOESM1_ESM.docx]

PKU: Parenting experiences and well being

Qualitative study: Topic guide for semi structured interview

Version 1, 24.04.2015

What follows is a guide:-

The order and exact content of the questions will be determined by the participant so the order and wording of the questions may vary as the interview develops.

The following topics and prompts serve as an interview guide.

Probe and ask for examples as the time permits.

Introductions & Background

Explain purpose of interview. Reassure that the interview will not have an impact on the care they receive and that they are free to stop the interview at any time.

This statement will be read to the participants:

“As parents you are adjusting to finding out that your child has been given a diagnosis of PKU. Since that time, you might have been thinking about what that means not only for your child, but also for you as a parent. We are interested in finding out about what it is like to be a parent to a child with PKU and the parenting process. By parenting we mean promoting and supporting the physical, social and emotional development of your child. We understand that the dietary aspect of parenting is very important, however being a parent includes many more things. We are interested in finding out more about the impact a diagnosis of PKU might impact on some of these things. We have some loosely structured questions, however we want to find out what has been important to you in your experiences so far. If you have more than one child with PKU we would like to focus on the child who is under the age of 2”.

Diagnosis of PKU

- Explore parent’s knowledge and understanding of PKU prior to their child being diagnosed (for example if this is not their first child with PKU was this different this time).

The process of parenting

- Explore experiences of parenting (child) so far
- Explore what parenting (child) involves (getting an idea about the demands, how much time can be dedicated to other parenting roles alongside dietary management, thinking about emotional, social, physical development as well as bonding with child).
- Description of the way the parent feels they parent their child (parental styles- strict, permissive etc)
- Explore how the diagnosis might have changed the way in which they thought they would parent their child (adaptations to parenting)
- Any impact this has had on how they interact with their child
- Differences in parenting other children (if they have other children who are older and have PKU, or do not have PKU)
- Exploration of how parents perceive role in parenting (how they see their ‘job’ as parent)

Challenges

- Find out if there have been times when parenting their child has been hard (specific challenges- e.g. weaning)
- Asking about expected and unexpected challenges that have arisen in parenting (physical, social, emotional).
- Exploring how parents have managed these (emotionally, physically?)
- Exploring any positives/positive experiences to date (related to PKU or parenting more generally)
- Any personal feelings or thoughts that impact on parenting

Support

- Things that have helped with parenting child
- Thing/experiences that have helped parent personally/ not helped (things that they do themselves)
- Things that others have done that have/have not helped? (professionals, family members, friends)
- Experience of the support has been provided.

**Any other important topics?**

- Check with participant whether there are any other important aspects that have not been covered

Following interview

- Thank participant.
- Ask participants how they have found the interview.
- Clarify whether participant has experienced any distress above what might be expected (due to discussing sensitive issues).
- Researcher to give participant contact details of support organisations and signpost participant to appropriate agencies if they are distressed.
